# Middle-shallow feature aggregation in multimodality for face anti-spoofing

**DOI:** 10.1038/s41598-023-36636-w

**Published:** 2023-06-19

**Authors:** Chunyan Li, Zhiyong Li, Jianhong Sun, Rui Li

**Affiliations:** Engineering College, Honghe University, Mengzi, 661100 Yunnan China

**Keywords:** Development of the nervous system, Neural patterning, Electrical and electronic engineering

## Abstract

At present, most advanced algorithms for face anti-spoofing use stacked convolutions and residual structure to obtain complex characteristics of deep networks, and then distinguish liveness and deception. These methods ignore the shallow features that contain more detailed information. As a result, the model lacks sufficient fine-grained information, which affects the accuracy and robustness of the algorithm. In this paper, we use the simple features of the shallow network to increase the fine-grained information of the model, so as to improve the performance of the algorithm. First of all, the shallow features are spliced to the middle layer by "shortcut" structure to reserve more details for the middle layer features and improve their detail representation ability. Secondly, the network is initialized with the best pre-trained model parameters under unbalanced samples, and then trained on the balanced samples to improve the classification ability of the model. Finally, RS Block based on depthwise separable convolution is used to replace res module, and model parameters and floating point operations are reduced from 18G and 61 M to 1.9 M and 347 M. The algorithm is simulated on CASIA-SURF dataset, and the results show that the average classification error rate (ACER) is only 0.0008, TPR@FPR = 10E−4 reaches 0.9990, which is better than the previous face anti deception methods.

## Introduction

Face recognition is widely used in face payment, device unlocking, access control and other applications, but it has security vulnerabilities. It is impossible to distinguish whether the face image on the imaging device is a real living person or a deception attack (such as printed photographs, video replays, 3D masks and others). Therefore, in order to prevent forged data from passing authentication, it is very important to design a face anti deception algorithm with high detection accuracy, high robustness and strong generalization ability ^1^.

At present, face anti deception is a very advanced research and application in the face field, and has gradually developed into a relatively independent research field. Anti deception algorithm is generally considered as a dichotomous problem. In the early stage, researchers used manually designed features (such as LBP, hog, surf) to identify real and deceptive faces ^2^. Yang et al.^3^ uses Convolutional Neural Networks (CNN) to extract features automatically for the first time, and this algorithm has obtained good results in the test. Since then, most anti spoofing algorithms use CNN, which is learned in a data-driven manner, to distinguish between live and spoofing attacks.

Neurons in high layers strongly respond to entire objects while other neurons are more likely to be activated by local textures, patterns, etc. ^4^. Therefore, various methods based on CNN extract high-level features with rich semantic information by building a multi-layer network, so as to identify whether the target is a living body ^5^. However, if all samples are classified according to complex features, the efficiency of the network will be greatly reduced. For example, in two category classification experiment to distinguish pure water from beverages, we can first look at the simple feature of liquid color. If there is color, it is a beverage. If there is no color, we can further judge whether it has taste. Since the simple feature of color can distinguish most samples, why should we take time to continue to test the taste of the liquid. Therefore, the shallow features of the network contribute to the classification.

However, after dozens or even hundreds of convolution operations, the shallow features are gradually lost, and there are few simple features left in the deep layer of the network. Zhao et al. ^6,7^ introduced the characteristics of shallow and deep features, and adopted different methods to build a feature pyramid structure. Liu et al. ^4^ considered the importance of shallow features in instance segmentation and pixel level classification, and proposed a bottom-up path enhancement method.

In this paper, we use the "shortcut" structure to design the model, and do a lot of experiments on the CASIA-SURF dataset. The results show that the effect of this model has reached the highest level, in which ACER = 0.0008, TPR@FPR = 10E−4 = 0.9990. The contributions of this paper are summarized as follows:

- The shallow features of the network are directly transmitted to the middle layer through the "shortcut" structure. The aggregated middle layer features effectively retain the resolution of shallow features and improve the detail representation ability of single mode features.
- The model is initialized with the best pre-trained model parameters under unbalanced samples, and then trained on the balanced samples to improve the classification performance of the model.
- RS Block based on depthwise separable convolution(DSC) is used to replace res module, which greatly reduces network parameters and computation. The parameters and FLOPs of this model are only 0.03 times that of^8^.

## Related work

From the perspective of the development history, the face anti deception algorithm mainly goes through two stages: manual feature stage and CNN based stage. The algorithm based on manual features ^9–13^ is sensitive to lighting, photographic equipment and other conditions. In 2015, Wen proposed a live face detection algorithm based on image distortion features, taking advantage of the different distortion features of real and fake face images. However, this method has achieved little with the emergence of high-resolution cameras and printing equipment.

The methods based on CNN can learn more general features, which is more advantageous than manually designed features, and is conducive to the improvement of algorithm robustness and generalization ability. ^1,5,14,15^ used the DSC to build neural network and distinguished the deception attack from the real face by the extracted features. Yu et al. ^16^ extracted more detailed information using the central difference convolution, which improved the performance of the model. ^1,17^ integrated multimodal information in the middle layer of the network, and obtained the deep network information for classification through subsequent module processing. ^8^ simultaneously used two feature fusion methods : mid-level and high-level feature fusion. The model not only aggregated multimodal features at the same level, but also fused multimodal features at the middle level, and finally fused these two features at the deep level of the network.

The datasets used for anti spoofing algorithms include CASIA-FASD, MSU-MFSD, Siw and CASIA-SURF. The CASIA-FASD, MSU-MFSD, and Siw datasets contain only single mode images (RGB), while CASIA-SURF contains three modes (RGB, IR, and Depth). Although RGB images can describe the contour, color and other information of objects, with the emergence of various new deception attacks and high-resolution printers, a single mode image is not enough to provide a high level of security. In fact, each mode of image has unique characteristics and advantages. For example, infrared cameras are not sensitive to electronic displays, so IR is effective against playback attacks. The depth image can describe the spatial geometric information of the object, which can effectively resist the printing attack. Therefore, multimodal images can improve the performance of the anti deception algorithm. The CASIA-SURF dataset ^18,19^ has broken through the limitation of living detection tasks in terms of quantity and image type. It has three modes (RGB, IR and Depth), including 21,000 videos of 1000 subjects. Each subject contains one real video and six fake videos, and each fake video corresponds to different types of print attacks.

A most related work to ours is ^8^, which introduces a multi-modal face anti deception method -MLFA. MLFA uses ResNet 34 and ResNet-50 as the backbone of the network, and uses both mid-level and high-level feature fusion. It uses three branches composed of res modules to extract middle-level features of different modes respectively, and uses agg module to integrate multimodal features of the same level. These features are fused together at the high level of the network to form multi-level fine features of the model. The structure of MLFA is very complex, including 4 res and 3 agg modules. Therefore, MLFA not only has complex algorithms, but also has a large amount of computation. The parameter of MLFA is as high as 61 M, and the computational complexity is 18G.

Differently from the baseline method, we first remove the agg module, which greatly simplifies the complexity and computation of the model. Secondly, we use the "shortcut" structure to send shallow features to the middle layer of the network and aggregate them, so that the single-modal features in the middle layer can retain more detailed information. This improves the detailed representation ability of features. Thirdly, we initialize the network using the optimal pre-trained model parameters under imbalanced samples, and then train the model on balanced samples. The experimental results show that the performance of this method is superior to that of the random initialization method. Finally, we use RS Block based on DSC and inverse residual structure to replace the res module. Compared with the res module based on ordinary convolution, RS Block can significantly reduce network parameters and computation. By using the "shortcut" structure, we effectively utilize the shallow features of the model and improve the detailed representation ability of the middle features; In addition, the removal of the agg module and the use of depthwise separable convolution greatly reduce model parameters and computational complexity. Our Params and FLOPs are only 0.03 times that of ^8^, while the average classification error rate ACER of this model is only 0.0008, TPR@FPR = 10E-4 reaches 0.9990, which is superior to the method in ^8^.

## Methods

### Shortcut

**Figure 1:**
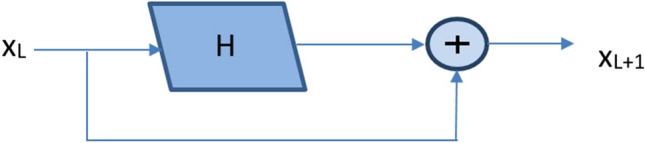
The structure of the residual module.

When the neural network propagates forward, each convolution layer can only extract a small part of the image information. With the deepening of the model, the network can obtain more complex feature patterns, but also lose a lot of original image information. The addition of identity mapping in the residual module ensures that the network of layer L + 1 must contain more image information than that of layer L. The structure of the residual module is shown in Fig. 1, and the formula is:

1 $${\text{x}}_{{{\text{L}} + {1}}} = {\text{x}}_{{\text{L}}} + {\text{H}}({\text{x}}_{{\text{L}}} ,{\text{w}}_{{\text{L}}} )$$

The residual module (x_L+1_) consists of the identity mapping (x_L_) and the residual mapping H (x_L_, w_L_). H (x_L_, w_L_) is generally composed of two or three convolutions.

^20^ points out that it is not that the more complex the feature is, the more effective it is. On the contrary, it is the most effective when the feature complexity is half of the network depth. The middle layer features of neural network have very rich information, including simple feature components and complex feature components that can only be learned through complex transformation. The simple feature components are obtained through simple transformation at the shallow layer of the network, which contain more location and detail information. The complex feature components are obtained in the deep layer after multiple transformations. If the complexity of feature component is quantified by the minimum number of nonlinear transformations required for learning the feature component, the middle level feature can be expressed as:

2 $${\text{f}}_{{\text{m}}} \left( {\text{ x }} \right) \, = {\text{f}}^{{1}} \left( {\text{ x }} \right) \, + {\text{ f}}^{{2}} \left( {\text{ x }} \right) \, + {\text{ f}}^{{3}} \left( {\text{ x }} \right) \, + \ldots + {\text{ f}}^{{\text{K}}} \left( {\text{ x }} \right) \, + \Delta {\text{f}}$$

where f_m_ (x) is the middle layer feature, f^k^ (x) represents the feature components with different complexity, △ f is the feature component of higher order complexity.

**Figure 2:**
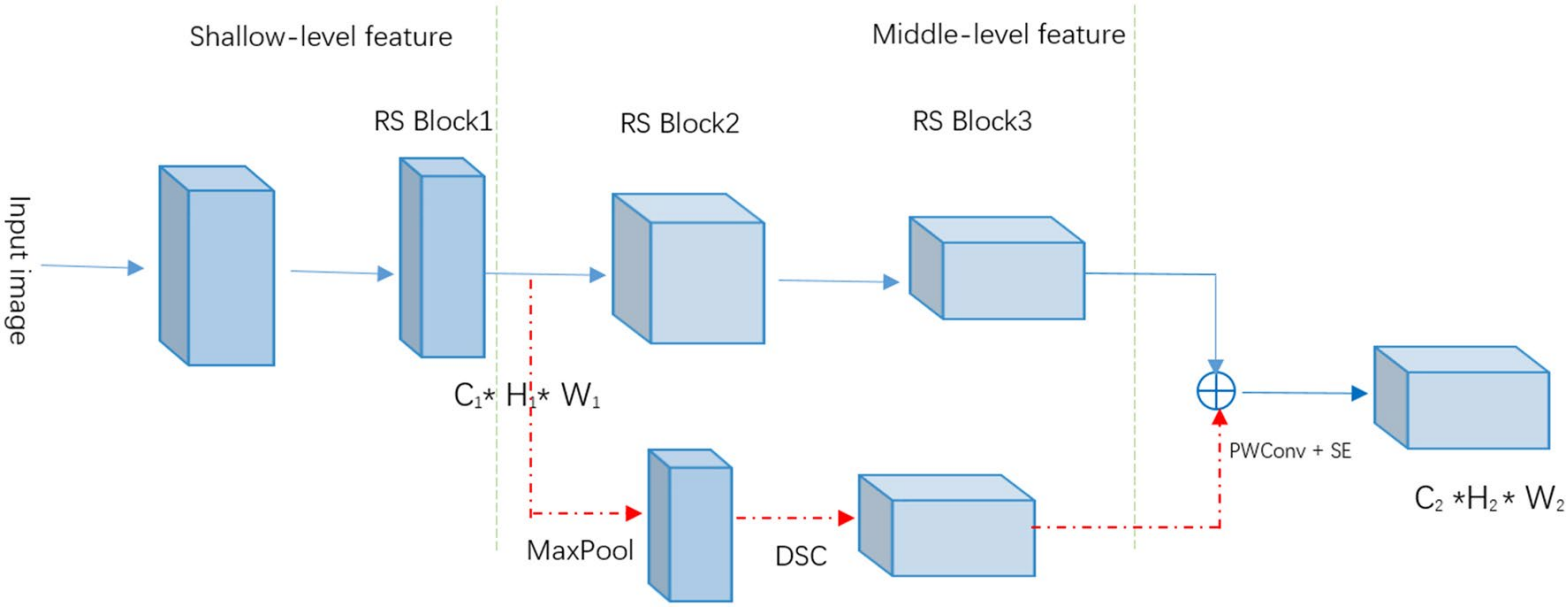
Modified shortcut.

In addition, the shallow features of neural network contain a lot of details such as edges, shape, location, etc. It has a high response to edges and instances, but it has low semantics and much noise. If the shallow features are directly transmitted to the deep layer of the network, the noise it contains will affect the classification effect of the model. In order to effectively utilize the shallow features, we use the "shortcut" structure to transfer it to the middle layer of the network. After concatenating with the middle layer features, they are squeezed and excited to obtain the weighted fusion features, as shown in Fig. 2. Finally, these features are fed into the deep layer of the network (RS Block4) for further processing. After multiple convolutions, the noise in the fused features gradually subsides, and the network finally outputs the deeper features with higher fine-grained information.

The shortcut structure is a double-layer residual structure, or a nested residual. The first layer residual is in the RS block, and each RS block is composed of n inverse residuals. These RS blocks in turn form the basic framework of the network. The second layer residual is created based on the first layer residual, which means it is built on the shallow layer (RS Block1) and the middle layer (RS Block3) of the network. It is worth noting that in the second layer of residual, identity mapping (X_L_) is replaced by maximum pooling and depthwise separable convolution (DSC), rather than directly using shallow features. This is because the output features of RS Block1 and RS Block3 are different in size and dimension, so they cannot be directly concatenated. In order to retain the shallow information as much as possible and ensure that the size and dimension of the shallow and middle features are consistent, this paper uses the combination of maximum pooling and DSC. After these two operations, the size of the shallow feature changes to the original 1/4, and the feature dimension is consistent with the middle feature. When using pooling with step size of 4 and 1 * 1 convolution, the size and dimension of the output feature can also be consistent with the middle-level feature, but this method loses more information.

The execution process of residual module is "compression—depthwise convolution—expansion", while the process of inverse residual is "expansion—depthwise convolution—compression". Expansion means to increase the dimension of features, while compression means to reduce the dimension of features. Both of them can be realized by 1 * 1 convolution. Depthwise convolution is used to extract features, but it does not change the number of feature channels. Therefore, if channel compression is performed on the input features first and then depthwise convolution is performed, the information loss of low-dimensional features will be serious after ReLU activation; On the contrary, if channel expansion is performed on the input features first and then the deepwise convolution is performed, the probability of the high-dimensional features distributed on the ReLU activation band is higher, which means that the information loss of high-dimensional features after ReLU activation is less. In view of this, MobileNetV2 replaces the residual module with inverse residual. In this paper, the inverse residual is used in the first level residual.

The shortcut structure combines the accurate positioning information in shallow layer with the information in the middle layer, realizes the aggregation of semantic information from the low layer to the middle layer, and increases the fine grained information of single mode features. In addition, the shortcut structure can transmit more shallow information to the deep layer of the network, solving the problem of gradient divergence and feature reuse in the deep network.

### Model structure

**Figure 3:**
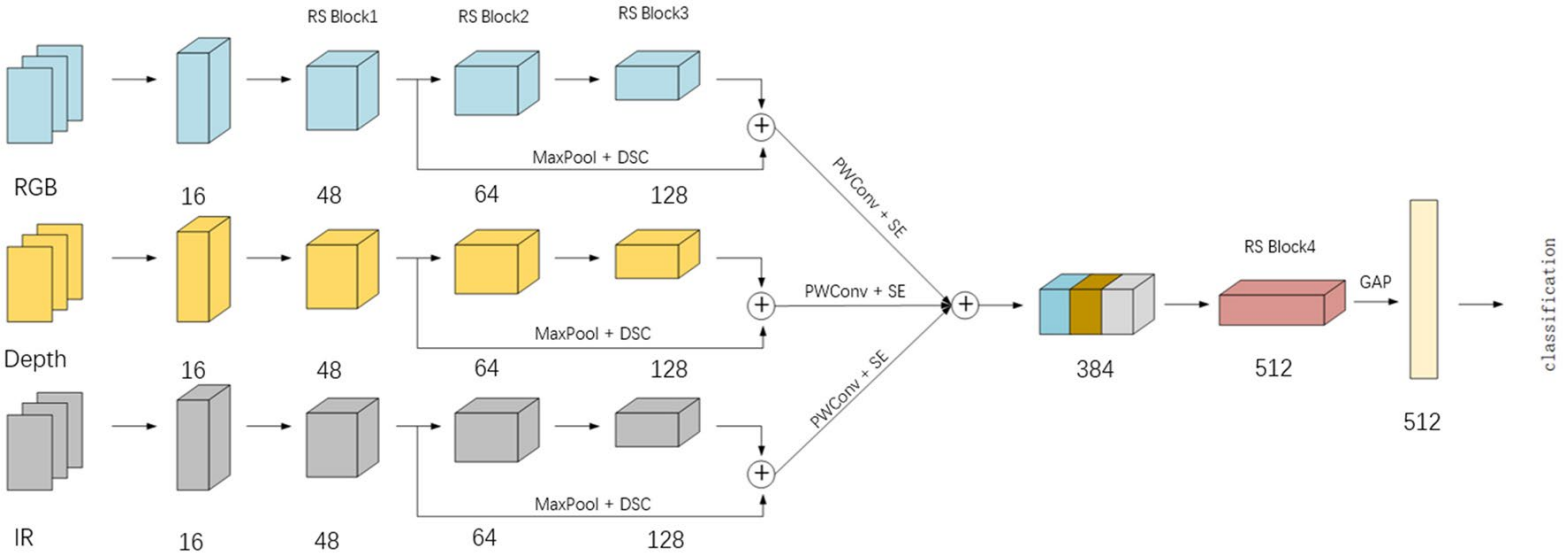
The proposed architecture.

The multimodal middle shallow feature aggregation (MSFA) model is shown in Fig. 3, which is built by several RS Blocks. RS Block is the backbone of the model and is responsible for extracting the feature of each layer. On the diagram: GAP—global average pooling; ⊕—concatenation.

The model has three branches with the same structure, which process data of three modes (RGB, Depth and IR) respectively. Each branch consists of 1 ordinary convolution and 3 RS Block modules. The shallow features of RS Block1 in the branch are spliced with the middle features of RS Block3 in the way of "shortcut". After squeezing and excitation, the fusion features of each mode are aggregated and fed into RS Block4. Finally, they are sent to the classifier for classification after global average pooling.

The first layer of the model uses ordinary two-dimensional convolution (Conv2d) , which can retain more features, while the remaining layers are constructed from RS blocks, as shown in Table 1. In the table, exp-expansion factor, n-the number of inverse residual modules, c-the number of channels. All point by point convolutions use 1 × 1 kernels, and the rest convolutions use 3 × 3 kernels.

**Table 1:** Network architecture.

Input	Operator	Exp	N	C
112^2^ × 3	Conv2d	–	–	16
56^2^ × 16	RS Block	1	3	48
28^2^ × 48	RS Block	6	2	64
14^2^ × 64	RS Block	4	3	128
7^2^ × 128	cat	–	–	384
7^2^ × 384	RS Block	1	2	512
3^2^ × 512	GAP	–	–	512

The RS block of the model sets different exp, n and c. Moreover, we use GAP to compress the feature maps to 1 × 1 × 512, which not only reduces the amount of calculation but also avoids the risk of overfitting caused by using full connections.

### RS block

**Figure 4:**
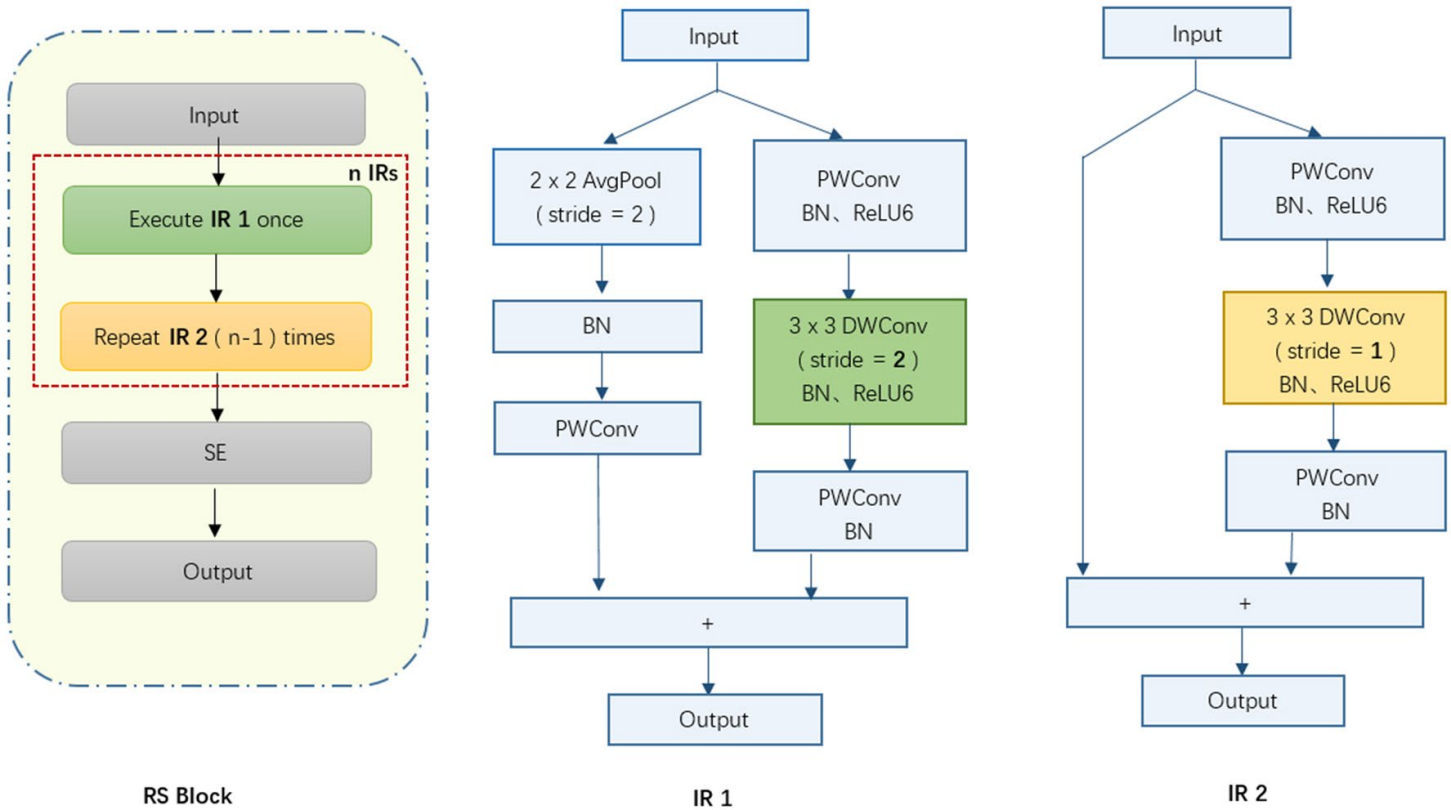
Composition of RS Block.

RS Block is the cornerstone of our network and is responsible for extracting features. It is composed of an indirect mapping residual(IR1), (n-1) direct mapping residual (IR2) and a lightweight spatial attention mechanism, as shown in Fig. 4. In IR1, the identity mapping is no longer x_L_, but downsampling, and the residual mapping is an inverse residual, where the step size of depthwise convolution is 2. Because the adjacent receptive fields are not repeated, the output size of the deepwise convolution is reduced to half of the original size, achieving a similar effect of "pooling". The downsampling module consists of average pooling (the step and kernel size are both 2), batch normalization and 1*1 convolution. ^21^ has proved that average pooling (AP) can embed multi-scale information and aggregate features of different receptive fields, which is beneficial for model performance. 1*1 convolution can add nonlinear excitation to the upper features and improve the expression ability of the network. IR2 uses the inverse residual block proposed in ^22^, that is, identity mapping + inverse residual. In the residual mapping, the step size of convolution is set to 1, so that there is a repeating area between the receptive field of adjacent steps. This enables feature extraction with higher accuracy.

RS blocks in different layers contain different inverse residuals and expansion rates. The expansion rate determines the "expansion" dimension in each inverse residual module. When performing deepwise convolution, the larger the expansion rate, the higher the dimension of the features. Generally, as the depth of the network deepens, the dimension of the feature gradually increases, while their size gradually decreases. The shallow features have low dimension and large size; The deep features have high dimensions and small size. Therefore, the expansion rate of shallow layer (RS Block1) and deep layer (RS Block4) of the model is set to 1. The middle-level feature contains very rich information. If it is expanded to high dimensions and then deepwise convolution is performed, more feature information will be obtained. Therefore, the expansion rate of the middle layer (RS Block2, RS Block3) is higher than that of the shallow and high layers. In addition, due to the easy optimization of the residual module, the RS Block adopts an inverted residual structure. After stacking the inverse residuals, a deeper network can be realized, which can gradually extract high-level features and increase the receptive field of the model. Through multiple comparative experiments, this article sets the number of inverse residuals and expansion rate among the four RS blocks as follows: (3, 1), (2, 6), (3, 4), and (2, 1).

### Lightweight spatial attention mechanism

**Figure 5:**
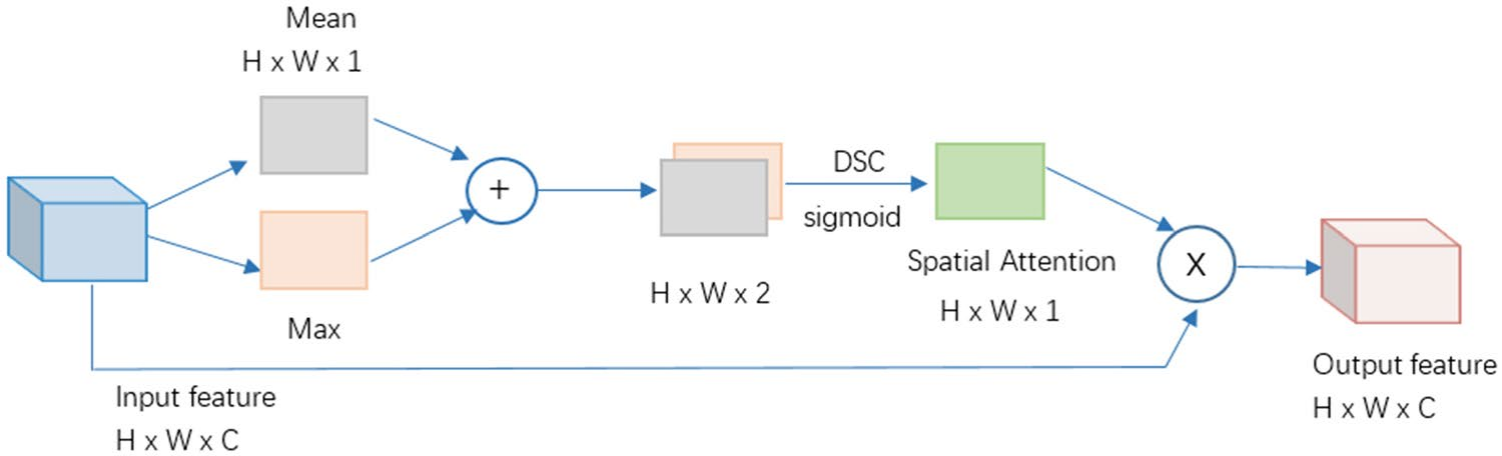
Lightweight spatial attention mechanism.

The lightweight spatial attention mechanism SE is used in both the model and RS Blocks, and its composition is shown in Fig. 5. Spatial attention mechanism is an adaptive spatial region selection mechanism, which can capture global information. The larger the eigenvalue in the feature map, the greater the variance of the matrix on the corresponding eigenvector, and the greater its power and information. This means that some specific features may be detected; The smaller the eigenvalues, the smaller the amount of information in these directions. Therefore, we extract the average and maximum values of the input features respectively on the channel dimension, and obtain two compressed channel features with the size of H x W × 1; After concatenation, dimension reduction, and activation, the feature weights for each position are obtained; Finally, they are multiplied by the input features to enhance the regions of interest and suppress useless information.

### Pre-trained model parameters

The samples in the CASIA-SURF dataset are extremely unbalanced: negative samples are six times larger than positive samples. If the model is trained with imbalanced samples, the fitness of negative samples is high, and the Attack Represents Classification Error Rate (APCER) is low. However, the Normal Represents Classification Error Rate (NPCER) is not ideal, which is caused by the low fitting degree of the positive samples. Therefore, for imbalanced samples with a large number of negative samples, the model parameters are more conducive to the classification of negative samples. Unbalanced training data may have a serious negative impact on the overall performance of CNN, while balanced training data will produce the best classification results ^17^. Given the above analysis, we first pre train the model using an imbalanced dataset and obtain the optimal training model parameters. Then we double the positive samples in CASIA-SURF and perform multiple data augmentation operations to obtain a balanced dataset. Finally, we initialize the network with the best pre trained model parameters under imbalanced samples, and then train the model on balanced samples. The results show that the effectiveness of this method is better than directly using random initialization.

## Experiment

### Evaluation metrics

For the performance evaluation, the commonly used metrics are: Average Classification Error Rate (ACER), Attack Represents Classification Error Rate (APCER), Normal Represents Classification Error Rate (NPCER), TPR@FPR = 10E−2, 10E−3, 10E−4. ACER is treated as the evaluation metric, in which APCER and NPCER are used to measure the error rate of fake or real samples respectively ^15^.

### Implementation details

The MSFA model we proposed is implemented by Pytorch and trained on two NVIDIA Tesla T4s. The model uses Adam optimizer and cosine learning strategy for training. The learning rate starts from 0.0002, and the batch size is 32. He Kaiming initialization method is used to initialize the model weight during pre-trained model. We perform random rotation, scaling, clipping and other preprocessing on the image during training.

The training set consists of three types of attack images (e_b, en_s, enm_b) and real faces. The verification set consists of images of other attack types (en_b, enm_s) and real faces. To test the generalization capability of the model, we set up five groups of test sets to verify the model. They are test1 (en_s, enm_s), test2 (enm_s, enm_b), test3 (e_s, en_b, enm_s), test4 (e_b, en_s, enm_s), and test5 (e_s, e_b, en_s, en_b, enm_s, enm_b).

### Result analysis

#### Advantages of the shortcut

In this paper, the shallow features are sent to the middle layer of the network by "shortcut", increasing the simple feature components of the middle layer features. In order to verify the effectiveness of this method, we train the baseline method (MLFA) and the proposed model(MSFA) on the same train set and validation set respectively, and test them on the same test set. Its values of ACER and TPR@FPR is shown in Table 2. It can be seen from the table that the ACER of MSFA is lower than that of MLFA, the values of TPR@FPR = 10e-4 (except the values of the third and fourth groups) are higher than the MLFA, and the overall performance of MSFA is better than the MLFA. MSFA uses the "shortcut" structure to transmit shallow information to the middle layer of the network, which adds more detailed information to the middle layer of the network. After further processing, the model obtains advanced features that contain more fine-grained information. Therefore, the accuracy and robustness of the MSFA algorithm are higher.

**Table 2:** Performance comparison of two models.

Test	MLFA		MSFA	
	ACER	TPR@FPR = 10e−4	ACER	TPR@FPR = 10e−4
test1	0.00567	0.954	0.00198	0.989
test2	0.00578	0.954	0.00208	0.946
test3	0.01270	0.882	0.00476	0.873
test4	0.01262	0.905	0.00278	0.961
test5	0.01282	0.902	0.00389	0.912

Moreover, we conduct another experiment to verify the effectiveness of the "shortcut". We delete the "shortcut" structure in MSFA and keep the remaining structure of the model unchanged, and train and test it. Table 3 shows the ACER and TPR@FPR Value.

**Table 3:** Performance analysis of "shortcut" structure.

Test	MSFA		MSFA (without shortcut)	
	ACER	TPR@FPR = 10e-4	ACER	TPR@FPR = 10e-4
test1	0.00198	0.989	0.00516	0.992
test2	0.00208	0.946	0.00678	0.992
test3	0.00476	0.873	0.01403	0.000
test4	0.00278	0.961	0.01016	0.898
test5	0.00389	0.912	0.01270	0.000

The experimental results show that the ACER values of the model wihtout shortcut are higher, and the TPR@FPR = 10e−4 values are not ideal on some test sets. In the middle layer of the network, model without "shortcut" only contains middle layer features, which include both simple and complex feature components. As the subsequent processing progresses, the simple feature components in the middle level features gradually transform into complex feature components after multiple transformations, which means that the simple feature components gradually disappear. Therefore, the high-level features extracted by the model lack sufficient fine-grained information, which reduces the classification ability of the model.

#### Compare with other network performance

As shown in Table 4, experiments are executed to compare with other network’s performance. All experimental results are based on CASIA-SURF images, and then the performance is verified on the CASIA SURF validation set. Both MSFA and MLFA use three branches to fuse multimodal data (IR, RGB, Depth). Compared with these models, our proposed model achieves better performance on the validation set. This indicates that the four improvement measures we have taken for the model are effective. That is: ① Delete the AGG module; ② Use the "shortcut" structure; ③ Initialize the network using the optimal pre trained model parameters under imbalanced samples; ④ Use RS Blocks instead of the res modules.

**Table 4:** Performance comparison of multiple models on validation set.

Model	ACER	TPR@FPR = 10E−2	TPR@FPR = 10E−3	TPR@FPR = 10E−4
MSFA	0.00079	1.0000	0.9997	0.9990
Baseline	0.00158	0.9999	0.9976	0.9884
MobilenetV2	0.01245	0.9850	0.94367	0.85467
FishNet	0.02362	0.9610	0.9010	0.7490

#### Ablation experiments

**Figure 6:**
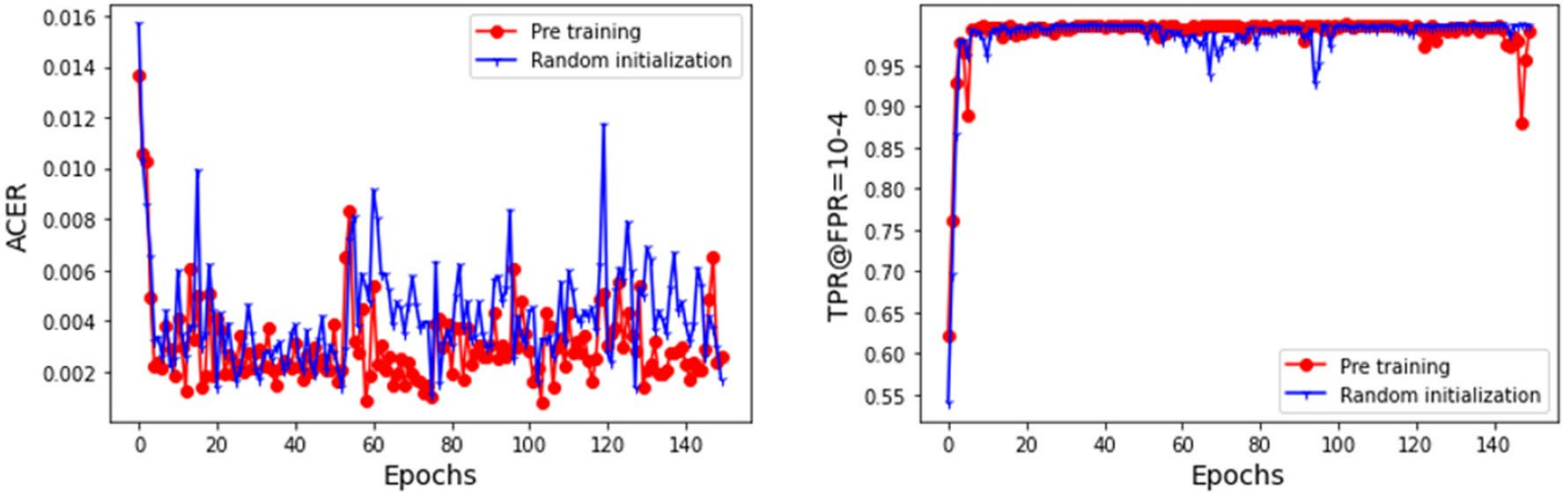
Performance comparison between random initialization and pre-trained model initialization.

*Pre-trained* To improve the performance of the model, we train the model on a dataset with imbalanced samples, then initialize the network with the optimal pre trained model parameters, and finally train the model on balanced samples. In order to verify the effectiveness of the proposed method, we keep the model structure and other hyperparameter unchanged, use He Kaiming initialization method to initialize the network, and then train the model on balanced samples. The comparison of training results between the two methods is shown in Fig. 6. From the figure, it can be seen that the proposed method can greatly improve model performance and achieve nearly perfect ACER: 0.00078971 and TPR@FPR = 10–4: 0.9990. This is because the samples in the CASIA-SURF dataset are extremely imbalanced: negative samples are six times larger than positive samples. When using imbalanced samples to train the model, the fitting of negative samples is high, and the Attack Represents Classification Error Rate (APCER) is low. Therefore, the model parameters of imbalanced samples are more conducive to the classification of negative samples. Unbalanced training data may have a serious negative impact on the overall performance of CNN, while balanced training data will produce the best classification results ^17^. In view of this, this article initializes the network with the best pre-trained model parameters under imbalanced samples, and then trains the model on balanced samples to achieve better results.

**Figure 7:**
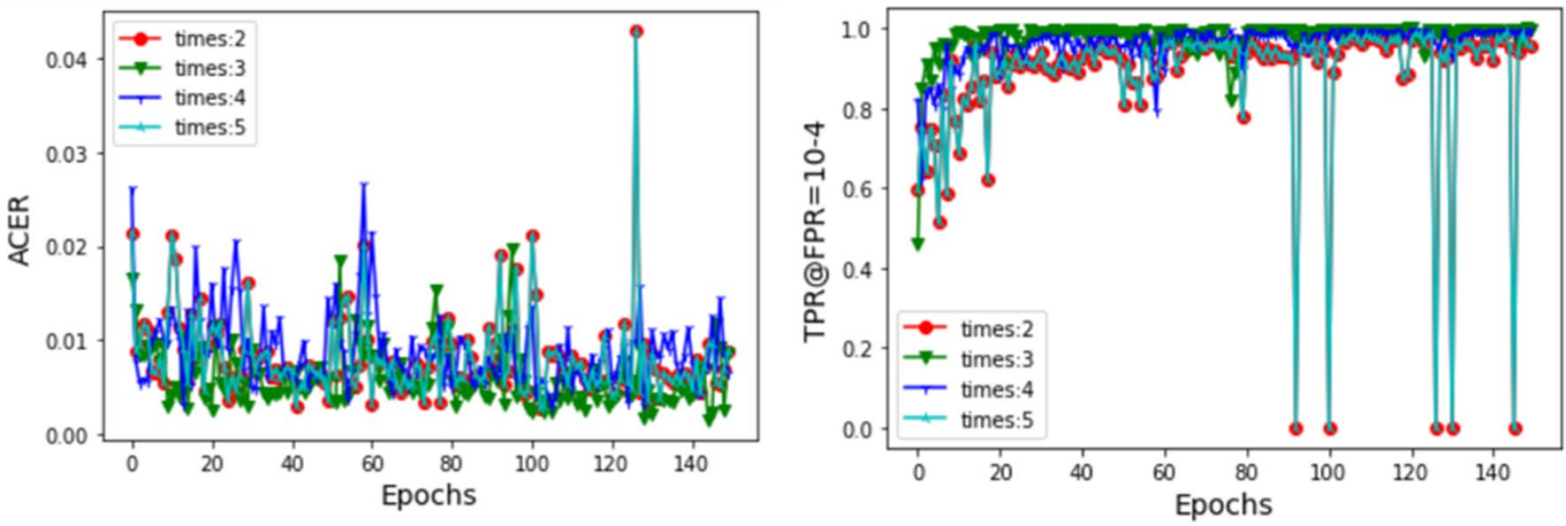
Performance comparison of different parameter settings.

*Determine the depth of shallow features* The receptive field of shallow network is small, and the overlapping area is also small. Therefore, the features extracted by shallow network are close to the input, including more pixel information (such as image color, texture, edge, corner, etc.) and noise information. Convolution can cause loss of detailed information, but it can reduce noise. So, how many convolutions does the model need to perform for shallow features? We set the number of IR in RS Block1 to 2, 3, 4, and 5 respectively, and train the model to analyze its performance, as shown in Fig. 7. The experimental results show that when the number of IR in RS Block1 is set to 3, its average classification error rate is generally better than the others.

After aggregating these shallow and middle features, MSFA needs to perform three convolution operations to obtain advanced features. When the number of IR is set to 2, shallow features contain relatively more noise. When the number of IR is set to 4 or 5, shallow features lose more detailed information after multiple convolution operations. From Fig. 7, it can be seen that shallow features with more noise or less detailed information are not conducive to model classification. Therefore, this article sets the number of IR in RS Block1 to 3. The number of convolution operations is just right, which reduces some noise and preserves appropriate detail information for shallow features.

#### Model complexity

The MLFA model uses ResNet 34 and ResNet-50 as the network backbone to extract features from each layer. MLFA has a complex structure, including 3 fusion levels and 4 branches; In addition, the model uses a large number of ordinary convolutions, resulting in huge parameters and calculations. The proposed model MSFA removes the agg module from MLFA, reduces the number of branches and complexity of the model, and adds a "shortcut" structure in the network to retain more detailed information; Moreover, MSFA uses DSC in RS Block, which reduces model parameters and computational complexity. These improvements greatly reduce the complexity and calculation of the model, with the parameters and FLOPs only 0.03 times that of MLFA. The complexity comparison of the two models is shown in Table 5.

**Table 5:** Complexity comparison with the baseline method.

Model	Params	FLOPS
MLFA	61.03M	18.2G
MSFA	1.907M	346.9M

#### Multi-modality

**Figure 8:**
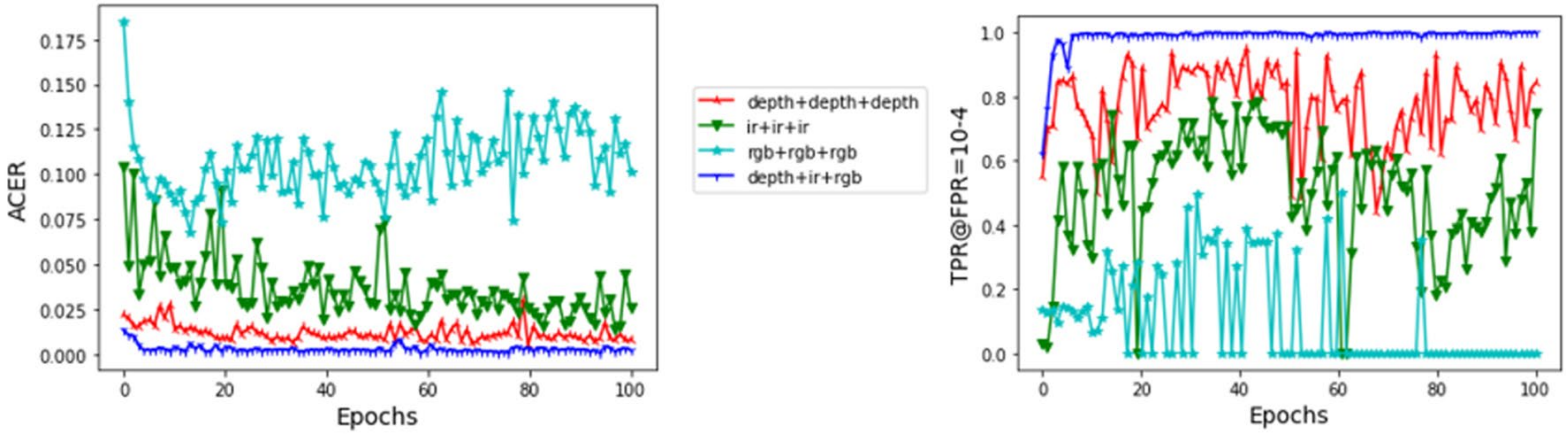
Performance comparison of single-modal and multi- modal networks on validation set.

We examine the advantage of multi-modal feature aggregation networks. With the model architecture and parameters unchanged, we replace the multimodal data (RGB, IR, Depth) of the three branches in MSFA with singlemodal data (RGB, RGB, RGB), (IR, IR, IR), and (Depth, Depth, Depth). We train them separately and their ACER and TPR@FPR are shown in Table 6 and Fig. 8.

**Table 6:** The effect of modalities measured on the validation set.

Modality	TPR@FPR			ACER
	= 10^–2^	= 10^–3^	= 10^–4^	
Depth + depth + depth	0.9977	0.9870	0.9313	0.00503371
IR + IR + IR	0.9840	0.8640	0.4810	0.01366487
RGB + RGB + RGB	0.7983	0.5758	0.3160	0.06776565
RGB + IR + depth	1.0000	0.9997	0.9990	0.00078971

From Table 6 and Fig. 8, it can be seen that the model using only depth images in single mode has the best performance, while RGB has the worst performance. However, the performance of single modal data lags far behind that of multimodal data. RGB images have high resolution, rich colors and textures, but are greatly affected by light; The depth information is invariant to the illumination change; The details and texture information of infrared image are fuzzy. Therefore, different types of single modal data have their own advantages and disadvantages. The multi-modal feature aggregation network can make full use of the advantages of each mode to generate the synergetic effect of modality fusion, which can significantly improve the performance of the algorithm.

## Conclusion

In this paper, we present a new method for face anti-spoofing detection which can improve the detail representation ability. It achieves the balance between algorithm performance and computational complexity. We discussed in detail three aspects: shortcut structure, initialization and depth of shallow features, and showed their significant improvements in improving the classification capability of the model. Firstly, We introduced the structure of the model, which consists of multiple RS blocks and uses a "shortcut" connection in the model. We have demonstrated that the shortcut structure can preserve more detailed information for the model and enhance the detailed representation ability of advanced features. Secondly, we compared the effects of random initialization and pre-trained model parameter initialization on the target task, indicating that using pre-trained model weights under unbalanced samples to initialize the network can improve the network performance. Thirdly, we conducted extensive comparative experiments to determine how many convolutions need to be performed in the shallow layer of the network to improve the classification ability of the model.

## Data Availability

The data that support the fingdings of this study are openly available in [https://github.com/hhxylcy3/Middle-shallow-Feature-Aggregation-in-Multimodality-for-Face-Anti-spoofing].

## References

[1] Deng, X., & Wang, H. Human face detection algorithm based on deep learning and feature fusion. *Comput. Appl*. http://www.joca.cn.2020:1009-1015.

[2] Tu, X., Zhao, J., Xie, M., et al. Learning generalizable and identity-discriminative representations for face anti-spoofing. arXiv preprint arXiv:1901.05602, Jan 2019

[3] Yang, J., Lei, Z., & Li, S.Z. Learn convolutional neural network for face anti-spoofing. arXiv preprint arXiv:1408.5601, Jan 2014

[4] Liu, S., Qi, L., Qin, H., Shi, J., Jia, J. Path aggregation network for instance segmentation. arxiv:1803.01534v4, 18 Sep 2018

[5] Pi, J., Yang, J., Yang, L., et al. Lightweight face detection method based on multimodal feature fusion. *Comput. Appl.* (2020)

[6] Zhao, G., Ge, W., Yu, Y. Graph feature pyramid networks for object detection. arXiv:2108.00580v3, 8 Jan 2022

[7] Zhao, Q., Sheng, T., Wang, Y., Tang, Z., Chen, Y., Cai, L., & Ling, H. M2Det: A single-shot object detector based on multi-level feature pyramid network. 10.48550/arXiv.1811.04533

[8] Parkin, A., & Grinchuk, O. Recognizing multi-modal face spoofing with face recognition networks. In *Proceedings of the 2019 IEEE Conference on Computer Vision and Pattern Recognition Workshops*, Piscataway, NJ: IEEE, 1617–1623 (2019).

[9] Yang, J., Lei, Z., Liao, S., & Li, S.Z. Face liveness detection with component dependent descriptor. In *2013 International Conference on Biometrics (ICB)*, 1–6. IEEE (2013)

[10] Patel K, Han H, Jain AK (2016). Secure face unlock: Spoof detection on smartphones. IEEE Trans. Inf. Forensics Secur..

[11] de Freitas Pereira, T., Anjos, A., De Martino, J. M., & Marcel, S. Lbp-top based countermeasure against face spoofing attacks. In *Asian Conference on Computer Vision*, 121–132. Springer (2012).

[12] de Freitas Pereira, T., Anjos, A., De Matino, J.M., & Marcel, S. Can face anti-spoofing countermeasures work in a real world scenario? In *2013 International Conference on Biometrics (ICB)*, 1–8. IEEE (2013).

[13] Boulkenafet Z, Komulainen J, Hadid A (2017). Face antispoofing using speeded-up robust features and fisher vector encoding. IEEE Signal Process. Lett..

[14] Howard, A., Sandler, M., Chu, G., et al. Searching for MobileNetV3[EB/OL]. 20 Nov 2019.https://arxiv.org/pdf/1905.02244.pdf.

[15] Zhang, P., Zou, F., Wu, Z., et al. FeatherNets: Convolutional neural networks as light as feather for face anti-spoofing. 22 Apr 2019. arxiv:1904.09290v1

[16] Yu, Z., Zhao, C., Wang, Z., Qin, Y., Su, Z., Li, X., Zhou, F., & Zhao, G. Searching central difference convolutional networks for face anti-spoofing. arXiv:2003.04092v1[cs.CV], 9 Mar 2020

[17] Shen, T., Huang, Y., & Tong, Z. FaceBagNet: Bag-of-local-features model for multi-modal face anti-spoofing. In *The IEEE Conference on Computer Vision and Pattern Recognition Workshops (CVPRW 2019)*, IEEE (2019).

[18] Zhang, S., Wang, X., Liu, A., et al. A dataset and benchmark for large-scale multi-modal face anti-spoofing. arXiv preprint arXiv: 1812.00408v3, 1 Apr 2019

[19] Zhang, S., Liu, A., Wan, J., Liang, Y., Li, S.Z. CASIA-SURF: a large-scale multi-modal benchmark for face anti-spoofing. arXiv preprint arXiv: 1908:10654v2, 4 Feb 2020

[20] Ren, J., Li, M., Liu, Z., & Zhang, Q. Interpreting and disentangling feature components of various complexity from DNNs. In *Proceedings of the 38th International Conference on Machine Learning*, PMLR 139,2021

[21] Szegedy, C., Liu, W., Jia, Y., Sermanet, P., Reed, S., Anguelov, D., Erhan, D., Vanhoucke, V., & Rabinovich, A. Going deeper with convolutions. In *Proceedings of the IEEE conference on computer vision and pattern recognition*, 1–9 (2015).

[22] Sandler, M., Howard, A., Zhu, M., Zhmoginov, A., & Chen, L.-C. Mobilenetv2: Inverted residuals and linear bottlenecks. In *Proceedings of the IEEE Conference on Computer Vision and Pattern Recognition*, 4510–4520 (2018).

[23] Paszke, A., Gross, S., Chintala, S., Chanan, G., Yang, E., DeVito, Z., Lin, Z., ban Desmaison, A., Antiga, L., & Lerer, A. Automatic differentiation in pytorch. 2017. 4

[24] Zoph, B., Ghiasi, G., Lin, T.-Y., Cui, Y., Liu, H., Cubuk, E.D., & Le, Q.V. Rethinking pre-training and self-training. arXiv:2006.06882v2[cs.CV], 15 Nov 2020

